# Combination value of cell index, CSF PCT, and CSF IL-6 for intracranial infection diagnosis after neurosurgery

**DOI:** 10.1038/s41598-025-97024-0

**Published:** 2025-04-13

**Authors:** Caimu Wang, Yiping Zhou, Chao Zhong, Hui Shan, Ping Wang, Zisheng Ge, Yushi Fan, Xinyun Zhang, Kai Zhang, Yesong Wang, Wei Cui, Linlin Du, Qijiang Chen, Gensheng Zhang

**Affiliations:** 1 Department of Critical Care Medicine, Ninghai First Hospital, Zhejiang, 315600 China; 2 Department of Critical Care Medicine, Second Affiliated Hospital, Zhejiang University School of Medicine, Hangzhou, 310009 China; 3 Department of Critical Care Medicine, Taizhou Hospital of Zhejiang Province, Zhejiang, 317000 China; 4 Key Laboratory of Multiple Organ Failure (Zhejiang University), Ministry of Education, Hangzhou, 310009 China

**Keywords:** Intracranial infection, Post-neurosurgery, Cell index, CSF PCT, CSF IL-6, Neuroscience, Medical research, Neurology

## Abstract

This study evaluated the predictive value of cell index (CI), cerebrospinal fluid procalcitonin (CSF PCT), and cerebrospinal fluid interleukin-6 (CSF IL-6) for detecting intracranial infection after neurosurgery. A two-center, prospective study analyzed CSF samples from ICU patients suspected of having intracranial infection following neurosurgery from January 2020 to June 2023. Patients with infection had longer operation times and longer stays in the ICU. The AUCs for single biomarkers ranged from 0.829 to 0.860, with the highest AUC of 0.938 observed for the combined biomarkers CI + CSF PCT + CSF IL-6. CI was most effective in patients with elevated CSF RBC counts, and PCT was most effective for detecting Gram-negative infections. The combined use of these biomarkers enhances early diagnosis of intracranial infection after neurosurgery and warrants further study.

## Introduction

Intracranial infection is one of the most common clinical complications after neurosurgery, with an incidence rate of 2.04–25%^1–3^. Once intracranial infection occurs, the mortality and disability of postoperative patients significantly increase^4–8^, along with a prolongation of hospitalization and an increase in hospitalization costs^9^. Therefore, it is very important to diagnose intracranial infection early and provide timely and effective treatment.

The clinical diagnostic criteria of intracranial infection after neurosurgery include characteristic clinical manifestations, imaging examination, blood test and general cerebrospinal fluid (CSF) test, while the etiological diagnostic criteria require positive microbiological test of CSF^10;11^. However, these diagnostic methods for intracranial infections have some limitations^10–14^ as follows: (i) Severe patients with consciousness disturbance make it challenging to evaluate clinical symptoms and signs; (ii) Traditional inflammatory indicators like white blood cells, neutrophil counts, and CRP lack specificity and sensitivity, making them difficult to differentiate between infection and inflammation; (iii) General examinations of CSF, including manometry, routine and biochemical tests, can be affected by mixed with blood after neurosurgery, leading to some difficulty in distinguishing infections; Although CSF culture is considered the gold standard, it is time-consuming with a low positive rate resulting in a delayed report with low sensitivity; (iv) Neuroimaging examinations can aid in diagnosis but may lack specific changes and pose some risk during transportation for critically ill patients; (v) Currently, metagenomic second-generation sequencing (mNGS) shows promise in pathogen detection but faces challenges like high cost, long waiting times, non-standardized testing processes, and disconnected interpretation of reports from clinical settings^15–17^. In addition, mNGS test is not available in some poor countries and regions. Overall, reliable early indicators for intracranial infection post-neurosurgery are still lacking, highlighting the need to explore some new methods with clinical significance.

CSF in patients after neurosurgery may experience blood contamination, leading to some alterations in its composition characterized by a notable rise in blood cells and proteins, which makes it insufficient/confused to diagnosis intracranial infection when assessing white blood cell count and neutrophil percentage. To address this limitation, the Cell Index (CI) calculated as the ratio of white blood cell count to red blood cell count in the CSF divided by the ratio of white blood cell count to red blood cell count in the blood is proposed, which has been shown to be helpful for the diagnosis of intracranial infection in patients with brain trauma or subarachnoid hemorrhage^18^. PCT levels in blood reflects systemic infection or inflammatory status, and has been extensively studied and applied in the diagnosis and treatment of various infections and inflammatory conditions. However, PCT levels in blood may not significantly increase in some localized infection lesions. Conversely, PCT levels in infected local body fluids offer a diagnostic advantage compared to PCT levels in blood^19–22^. Thus, CSF PCT might reflect more directly local condition of intracranial infection. Blood IL-6 reflects the extent of systemic infection or inflammatory states, making it valuable in assessing the severity of infection and systemic inflammatory response. In contrast, CSF IL-6, influenced directly by intracranial pathology, diminishes interference from systemic inflammatory response. Therefore, it typically exhibits higher specificity and sensitivity in diagnosing intracranial infections, rendering it more reliable compared to blood IL-6^23;24^. Therefore, CI, CSF PCT and CSF IL-6 are valuable in intracranial infection.

Herein, we designed this prospective clinical study to explore the feasibility and efficacy of CI, CSF PCT, and CSF IL-6 alone or their combination for the early prediction of intracranial infection occurence after neurosurgery, attempted to provide early daignosis and rapid treatment of intracranial infection.

## Materials and methods

### Study setting and participants

This two-center prospective case-control study was performed from January 2020 to June 2023 in the Second Affiliated Hospital, Zhejiang University School of Medicine and Ninghai First Hospital. Patients after neurosurgery with suspected intracranial infection who were admitted to the intensive care unit (ICU) were recruited in this study.

Exclusion criteria were as follows: (1) patients aged < 18 years; (2) pregnant women; (3) patients with other infection site besides intracranial infection; (4) patients with intracranial infection pathogen other than bacteria; (5) patients diagnosed as intracranial infection before the operation; (6) immunosuppressed patients with malignant tumor, or with immunodeficiency diseases, or long-term usage of hormones; (7) patients with serious dysfunction of heart, lung, or other important organs; (8) patients without pathogenic bacteria observed in the CSF sample; (9) patients with incomplete clinical data or withdrawal halfway; (10) informed consent form was unavailable.

### Definitions

The diagnosis of intracranial bacterial infection in patients after neurosurgery is defined by referring to the 2017 American Society of Infectious Diseases Clinical Practice Guidelines for Medical related Encephalitis and meningitis^10^, which were chosen for their comprehensive, evidence-based diagnostic and management approaches. The criteria for etiological diagnosis include meeting 1 or more of the following 1–4 items, and meeting item 5 to identify microorganisms. (1) Clinical presentation: Body temperature > 38℃ in the absence of another clear source of infection, with or without new headaches, meningeal irritation, seizures, and signs of worsening mental status; (2) Clinical imaging: CT or MR can show diffuse edema, dural thickening and strengthening or dilatation of the ventricular system; (3) Blood: white blood cells > 10 × 10^9^/L or neutrophil ratio > 80%; (4) The analysis of general characteristics of lumbar puncture and CSF meets one or more of the following: ① Lumbar puncture: CSF pressure can be increased (> 200 mmH_2_O, l mmH_2_O = 0.0098 kPa); ② General characteristics of CSF: CSF yellow, cloudy or typical purulent; ③ The total number of leukocytes in CSF was > 500 × 10^6^/L, and multinucleated cells > 80%; ④ CSF biochemistry: sugar < 2.8 mmol/L, protein > 0.45 g/L; (5) CSF microbial culture positive, or CSF metagenomic second-generation sequencing detected infectious microorganisms (All suspected intracranial infection patients underwent CSF culture and CSF metagenomic second-generation sequencing).

Clinical suspicion of intracranial infection after neurosurgery is defined as: meeting 1–2 of the following conditions at the same time. (1) Body temperature > 38℃ without other clear sources of infection, with or without new headache, meningeal irritation, seizures, signs of mental state deterioration; (2) White blood cells > 10 × 10^9^/L or neutrophil ratio > 80%, in serum.

### Data collection

All patient data including demographic data, clinical features, laboratory test results, CSF data, microbial related results, treatments and outcomes, were collected. The major laboratory test results included blood routine examination and serum biochemical test results, including renal and liver function, C-reactive protein (CRP), interleukin-6 (IL-6) levels and procalcitonin(PCT) levels measured within 24 h after suspected intracranial infection occurred. CSF was collected from ventricular drainage tube or lumbar puncture within 24 h of suspected intracranial infection, and CSF related tests including routine, biochemical, culture, mNGS, PCT and IL-6 were tested respectively. Acute Physiology and Chronic Health Evaluation II (APACHE-II) score and Sepsis Related Organ Failure Assessment (SOFA) score were calculated within 24 h after ICU admission. The primary outcome was 28-day mortality, and the secondary outcomes were length of stay in ICU, length of stay in hospital and 6-month Glasgow Outcome Scale (GOS) score.

### Statistical methods

The study hypothesized that a model combining CI, CSF PCT, and CSF IL-6 could effectively predict intracranial infections. The expected area under the ROC curve for this model was anticipated to exceed 0.7, with preliminary results indicating an AUC of 0.85. Using α = 0.05 (one-tailed) and β = 0.1, and a 1:3 case-to-control ratio, the sample size was estimated using PASS 15 software. It was determined that at least 35 patients with intracranial infections and 105 controls were required. Accounting for a 10% dropout rate, the study aimed to enroll 39 patients with intracranial infections and 116 controls.

SPSS 23.0 (IBM, Armonk, NY, USA) was used to statistically analyze the data. The figures were generated with GraphPad Prism 9 (GraphPad Software, CA, USA) and SPSS. All the measurements were initially tested for a normal distribution. Normally distributed data were presented as the mean ± standard deviation and were compared with the t-test, while non-normally distributed continuous variables were presented as the median and interquartile range (IQR) and compared using the Wilcoxon rank sum test. Categorical variables were reported as the number and proportion and were compared with the Chi-square test. To assess the discrimination, the Area Under the Curve (AUC) was calculated. A *P* < 0.05 indicates a significantly statistic difference.

## Results

### Admission and demographic data

**Fig. 1:**
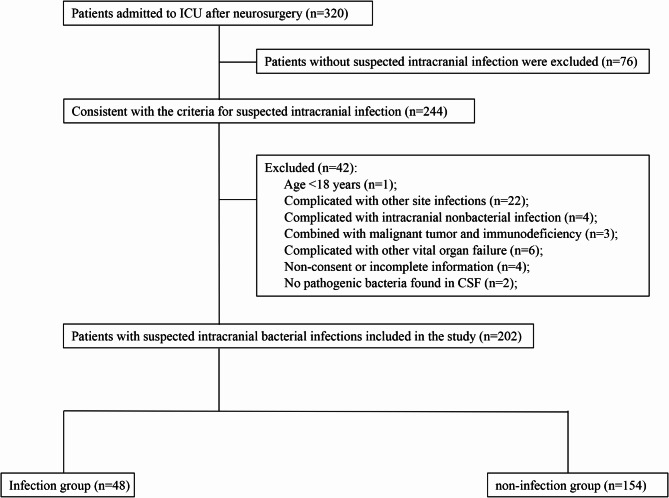
Flow diagram of participants enrolled in the study.

A total of 202 patients with suspected intracranial infection from initial 320 patients admitted to ICU after neurosurgery were finally recruited in the study (Fig. 1). These patients were aged 50.62 ± 14.58 years, with a male proportion of 52.48% (106/202). The occurrence of intracranial infection among these suspected patients was 23.76% (48/202). The duration of cumulative operation or drainage tube placement, and time from postoperation to suspected infection in intracranial infection group were significantly longer than those in intracranial non-infection group (all *P* < 0.05, Table 1). There were no statistical differences between the two groups in gender, age, BMI, comorbidities, APACHE-II score and SOFA score at admission (all *P* > 0.05) (Table 1).

**Table 1:** Clinical characteristics of patients with intracranial infection or non-infection after neurosurgery at admission.

	Intracranial infection group (*n* = 48)	Intracranial non-infection group (*n* = 154)	*P* value
Male (%)	26 (54.17)	80 (51.95)	0.788
Age (years)	50.67 ± 15.53	50.61 ± 14.33	0.981
BMI (kg/m^2^)	22.92 ± 2.4	22.62 ± 2.23	0.435
Comorbidities (%)	22 (45.83)	75 (48.70)	0.728
Hypertension (%)	17 (35.42)	58 (37.66)	0.779
Diabetes (%)	5 (10.42)	14 (9.09)	0.784
CVA (%)	2 (4.17)	4 (2.60)	0.576
CHF (%)	2 (4.17)	6 (3.90)	0.933
COPD (%)	2 (4.17)	5 (3.25)	0.761
CRI (%)	3 (6.25)	6 (3.90)	0.490
APACH-II score	16.94 ± 3.55	16.58 ± 3.26	0.514
SOFA score	6.40 ± 1.33	6.10 ± 1.23	0.161
GCS	6.04 ± 2.31	6.14 ± 2.23	0.785
Numbers of surgery	1.29 ± 0.54	1.2 ± 0.42	0.227
Cumulative operation time (hours)	4.24 ± 1.96	3.17 ± 1.16	0.000
Preventive use of antibiotics (n, %)	45 (93.75)	147 (95.45)	0.635
Number of drainage tubes	1.94 ± 0.81	1.73 ± 0.72	0.099
Drainage tube placement time (days)	5.79 ± 1.95	4.63 ± 1.52	0.000
Intrathecal injection of drugs	7 (14.58)	18 (11.69)	0.595
Time from postoperation to suspected infection (days)	6.63 ± 2.73	4.72 ± 1.82	0.000

CVA: cerebrovascular accident; CHF: chronic heart failure; COPD: chronic obstructive pulmonary disease; CRI: chronic renal insufficiency; APACHE-II: acute physiology and chronic health evaluation II score; SOFA: sepsis related organ failure assessment; GCS: Glasgow coma scale.

### The predictive value of CSF indicators and other indicators for intracranial infection after neurosurgery

**Fig. 2:**
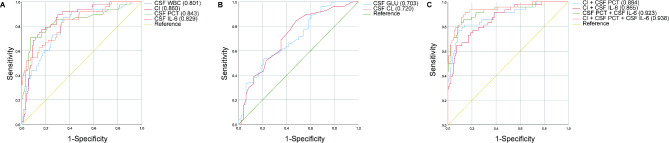
The predictive value of CSF indicators and other indicators for bacterial intracranial infection after neurosurgery. (**A**)ROC curves of CSF WBC, CI, CSF PCT and CSF IL-6. (**B**) ROC curves of CSF GLU and CSF CL. (**C**) ROC curves of CI + CSF PCT, CI + CSF IL-6, CSF PCT + CSF IL-6, and CI + CSF PCT + CSF IL-6.

On the first day of suspected intracranial infection, the detailed AUC areas to evaluate the predictive value of the occurrence of intracranial infection are shown in Table 2; Fig. 2. The AUC areas of CI (0.860), CSF PCT (0.843) and CSF IL-6 (0.829) were all larger than 0.7, which were larger than CSF Glu (0.703), CSF CL(0.720) and CSF WBC (0.801), respectively. Next, we suspect whether their combination would obtain a better predictive value. Indeed, the AUC area of CI combined with CSF PCT was 0.884, which was larger than that of CI (0.860) and CSF PCT (0.843), respectively; The AUC area of CI combined with CSF IL-6 was 0.865, which was larger than that of CI (0.860) and CSF IL-6 (0.829), respectively; The AUC area of CSF PCT combined with CSF IL-6 was 0.923, which was larger than that of CSF PCT and CSF IL-6, respectively. Of note, the most excellent AUC area was observed when these three indicators of CI, CSF PCT and CSF IL-6 were combined (0.938) (Table 2; Fig. 2).

**Table 2:** The predictive value of CSF indicators and other indicators for intracranial infection after neurosurgery.

	AUC(95%CI)	*P*	Cutoff	SE	SP	YI
CI	0.860(0.803–0.917)	0.000	4.202	0.771	0.838	0.608
CSF PCT (ng/ml)	0.843(0.770–0.916)	0.000	0.535	0.708	0.929	0.637
CSF IL-6 (pg/ml)	0.829(0.761–0.897)	0.000	5709.000	0.854	0.649	0.504
CSF Glu (mmol/L)	0.703(0.618–0.789)	0.000	3.230	0.792	0.532	0.324
CSF CL (mmol/L)	0.720(0.634–0.806)	0.000	119.500	0.521	0.844	0.365
CSF WBC (×10^6/L)	0.801(0.731–0.872)	0.000	76.000	0.875	0.649	0.524
sPCT (ng/ml)	0.581(0.484–0.677)	0.092	0.515	0.500	0.734	0.234
sIL-6 (pg/ml)	0.597(0.506–0.687)	0.043	28.500	0.750	0.442	0.192
sCRP (mg/L)	0.617(0.523–0.710)	0.015	18.750	0.521	0.792	0.204
CI + CSF PCT	0.884(0.825–0.943)	0.000	0.319	0.771	0.909	0.680
CI + CSF IL-6	0.865(0.807–0.923)	0.000	0.417	0.646	0.929	0.574
CSF PCT + CSF IL-6	0.923(0.875–0.970)	0.000	0.234	0.854	0.870	0.724
CI + CSF PCT + CSF IL-6	0.938(0.895–0.980)	0.000	0.167	0.917	0.857	0.774
CSF Glu + CI	0.845(0.785–0.905)	0.000	0.171	0.875	0.688	0.563
CSF Glu + CSF PCT	0.873(0.807–0.939)	0.000	0.285	0.771	0.896	0.667
CSF Glu + CSF IL-6	0.856(0.799–0.913)	0.000	0.352	0.688	0.877	0.564
CSF CL + CI	0.831(0.763–0.899)	0.000	0.210	0.771	0.773	0.544
CSF CL + CSF PCT	0.864(0.796–0.932)	0.000	0.308	0.729	0.909	0.638
CSF CL + CSF IL-6	0.857(0.791–0.924)	0.000	0.217	0.854	0.760	0.614
CSF WBC + CI	0.878(0.827–0.929)	0.000	0.150	0.938	0.721	0.658
CSF WBC + CSF PCT	0.901(0.847–0.955)	0.000	0.262	0.854	0.909	0.763
CSF WBC + CSF IL-6	0.854(0.789–0.920)	0.000	0.421	0.667	0.909	0.578
CSF Glu + CSF CL	0.762(0.683–0.841)	0.000	0.233	0.688	0.714	0.402
CSF Glu + CSF WBC	0.804(0.728–0.881)	0.000	0.218	0.729	0.760	0.489
CSF CL + CSF WBC	0.788(0.706–0.871)	0.000	0.270	0.646	0.903	0.548

CI: cell index, ratio between white blood cell count and red blood cell count in the CSF divided by the ratio between white blood cell count and red blood cell count; CSF PCT: cerebrospinal fluid PCT; CSF IL-6: Cerebrospinal fluid IL-6; CSF Glu: cerebrospinal fluid glucose; CSF CL: chloride ion in cerebrospinal fluid; sPCT: serum PCT; sIL-6: serum IL 6; sCRP: serum CRP; AUC: area under the curve; SE: sensitivity; SP: specificity; YI: Youden’s index.

### Subgroup analysis

**Fig. 3:**
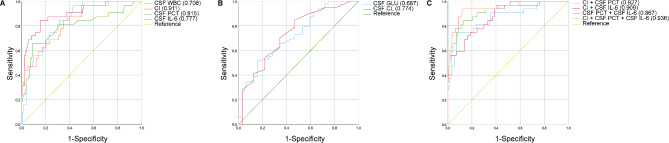
The predictive value of CSF indicators for bacterial intracranial infection after neurosurgery in the subgroup of CSF RBC > 3,000 × 10^6^/L. (**A**) ROC curves of CSF WBC, CI, CSF PCT and CSF IL-6, CSF WBC. (**B**) ROC curves of CSF GLU and CSF CL. (**C**) ROC curves of CI + CSF PCT, CI + CSF IL-6, CSF PCT + CSF IL-6, and CI + CSF PCT + CSF IL-6.

To further figure out whether these CSF indicators might obtain better predictive value in some specific conditions, we performed the subgroup analysis. In the subgroup of CSF RBC > 3,000 × 10^6^/L, 25 cases (21.93%) and 89 cases (78.07%) of these 114 cases had intracranial infection and no infection, respectively. The AUC area of CI (0.911) was larger than CSF PCT (0.815) and CSF IL-6 (0.777), respectively. The AUC area of the combined CSF indicators of CI + CSF PCT, CI + CSF IL-6, CSF PCT + CSF IL-6, and CI + CSF PCT + CSF IL-6 was 0.927, 0.909, 0.867, and 0.936, respectively(Fig. 3).

**Fig. 4:**
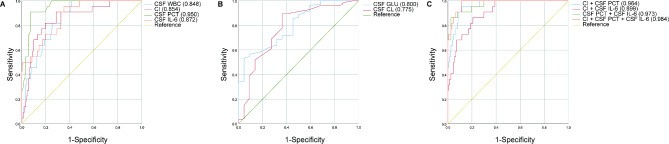
The predictive value of CSF indicators for Gram-negative bacterial intracranial infection after neurosurgery (single Gram-negative bacteria infection *n* = 22, no infection *n* = 154). (**A**) ROC curves of CSF WBC, CI, CSF PCT and CSF IL-6. (**B**) ROC curves of CSF GLU and CSF CL. (**C**) ROC curves of CI + CSF PCT, CI + CSF IL-6, CSF PCT + CSF IL-6, and CI + CSF PCT + CSF IL-6.

Among 48 patients in the infection group, there were 22 cases(10.89%) of single Gram-negative bacteria infection, 20 cases(9.90%) of single Gram-positive bacteria infection, and 6 cases of mixed infection with Gram-negative and Gram-positive bacteria. According to the subgroup analysis of Gram-negative bacterial intracranial infection, the AUC area of a single CSF indicator of CI, CSF PCT and CSF IL-6 was 0.854, 0.950 and 0.872, whereas it obtained 0.964, 0.899 and 0.973 when the combined CSF indicator of CI + PCT, CI + IL-6 and PCT + IL-6, respectively. Once CI, CSF PCT and CSF IL-6 were combined together, the AUC area obtained a perfect value of 0.984, (Fig. 4).

**Fig. 5:**
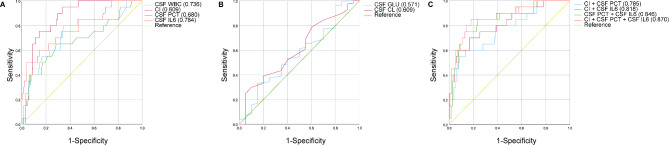
The predictive value of CSF indicators for Gram-positive bacterial intracranial infection after neurosurgery (single Gram-positive bacteria infection *n* = 20, no infection *n* = 154). (**A**) ROC curves of CSF WBC, CI, CSF PCT and CSF IL-6. (**B**) ROC curves of CSF GLU and CSF CL. (**C**) ROC curves of CI + CSF PCT, CI + CSF IL-6, CSF PCT + CSF IL-6, and CI + CSF PCT + CSF IL-6.

According to the analysis of intracranial infected subgroups of Gram-positive bacteria, 20 cases (9.90%) of Gram-positive bacteria were found. The AUC areas for single CSF indicators were as follows: CI, 0.865; CSF PCT, 0.680; CSF IL-6, 0.784. However, there was no significant increase in the AUC values when combining them with other CSF indicator combinations: CI + PCT (0.785), CI + IL-6 (0.818), PCT + IL-6 (0.846), and CI + PCT + IL-6 (0.870) (Fig. 5).

### Prognosis

The length of stay in ICU in the infection group was significantly longer than those in the non-infection group (21.02 ± 7.82 vs. 17.91 ± 7.35 days, *P =* 0.012 ), and the 6-month GOS score was significantly lower than that in the non-infection group (2.88 ± 1.02 vs. 3.42 ± 1.05, *P =* 0.002) (Table 3).

**Table 3:** Prognosis of patients with intracranial infection or non-infection after neurosurgery.

	Intracranial infection group (*n* = 48)	Intracranial non-infection group (*n* = 154)	*P* value
28-day mortality (%)	14.58% (7/48)	5.84% (9/154)	0.050
Length of stay in ICU (days)	21.02 ± 7.82	17.91 ± 7.35	0.012
Length of stay in hospital (days)	35.98 ± 21.96	30.94 ± 19.16	0.126
6-month GOS score*	2.88 ± 1.02	3.42 ± 1.05	0.002

*GOS score: Glasgow Outcome Scale score.

Although there was no statistical difference, the 28-day mortality was more than two-fold higher than that in the non-infection group (14.58% vs. 5.84%, *P =* 0.05), whereas the length of stay in hospital was similar between these two groups (all *P* > 0.05,Table 3).

## Discussion

The incidence and mortality of intracranial infection after neurosurgery are relatively high, and clinical diagnosis of intracranial infection is also challenging. In our study, several novel findings are shown below: ① the occurrence of intracranial infection among suspected patients was 23.76% (48/202). ② Among all suspected patients, the AUC for CI, CSF PCT and CSF IL-6 was larger than that for CSF Glu, CSF CL, and CSF WBC, respectively; The combined AUC of any two indicators was larger than that of any single indicator, with the largest AUC observed when combining CSF PCT with CSF IL-6. Excellent AUC was observed when all these three indicators of CI, CSF PCT, and CSF IL-6 were combined. Subgroup analysis found that where CSF RBC was > 3,000 × 10^6^/L, the AUC was largest for CI as an individual indicator, and the largest AUC was observed for CI combined with CSF PCT among these pairwise combinations. In the subgroup of intracranial infections caused by Gram-negative bacteria, the AUC was largest for CSF PCT as an individual indicator, and the largest AUC was observed for CSF PCT combined with CSF IL-6 among pairwise combinations. For Gram-positive bacterial intracranial infections, CI exhibited the largest AUC as an individual indicator, whereas there was no remarkable increase in the AUC when combined with these two or three CSF indicators. ③ Additionally, we found that intracranial infection led to a longer length of stay in the intensive care unit (ICU) and lower 6-month Glasgow Outcome Scale (GOS) scores.

In current study, 320 patients were admitted to the ICU following neurosurgery. After applying the inclusion and exclusion criteria, 202 patients with suspected intracranial infections who have conducted CSF test were enrolled. Finally, the number of patients with confirmed intracranial infections was 48, thus the incidence of intracranial infections in patients after neurosurgery was 23.76% (48/202). Although the occurence of intracranial infection after neurosurgery in our current study is in the range from 4.6 to 25% reported in previous study^3^, it still seems relatively high. Some factors might explain this high incidence as follows: ① High proportions of patients with a GCS score of ≤ 10 at the time of emergency presentation(96.04%, 194/202), with open head injuries (64.85%, 131/202), with re-operation within 48 h after the first surgery (20.79%, 42/202) and with comorbidities (44.55%, 90/202) were observed among those admitted to ICU with suspected intracranial infections. Thus, these more serious and complex situation in our current patients predisposed them to a higher risk of intracranial infections after neurosurgery; ② the average duration of surgery was 3.42 ± 1.46 h, and 94.06% (190/202) of patients required intracranial drain placement, which might further increase the risk of intracranial infection; ③ the extended duration of intracranial drain placement (4.91 ± 1.70 days) was identified as a significant risk factor for intracranial infection following neurosurgery.

In the current study, the diagnostic performance of CI, CSF PCT, and CSF IL-6 was observed to be superior to conventional CSF inflammatory markers such as CSF Glu, CSF CL, and CSF WBC in patients with suspected intracranial infection. This finding aligned with previous research results. CSF Glu, as an indicator of intracranial infection and inflammatory conditions, was susceptible to various factors affecting glucose metabolism and utilization. Changes in CSF CL could reflect the electrolyte balance of the CSF with lower specificity in diagnosing intracranial infections, while CSF WBC counts were influenced by blood contamination^10;25;26^. In contrast, CI reduced the interference from blood contamination^18^, CSF PCT exhibited high specificity and sensitivity for bacterial infections^19;20^, and CSF IL-6 demonstrated good sensitivity in early detection of intracranial infections^23;24^. While each of these markers held unique diagnostic value individually, they could also be affected by factors such as trauma or bleeding, limiting their utility as single indicators^10;25;27^.The combined use of these markers often enhanced diagnostic capability beyond that of any single marker. Specifically, the combination of CSF PCT and CSF IL-6 showed the greatest pairwise diagnostic potential, while the combination of CI, CSF PCT, and CSF IL-6 surpassed any pairwise combination of these markers. These findings not only underscored the importance of individual markers in diagnosing intracranial infections but also provided a theoretical basis for their combined application, aiding in the optimization of diagnostic strategies and treatment decisions in clinical practice.

In our study, we focused on patients with CSF RBC counts exceeding 3,000 × 10^6^/L on the first day post-operation, and CI demonstrated a superior diagnostic capability with an AUC of 0.911. This performance notably surpassed that of CSF PCT(0.815), CSF IL-6 (0.777), and CSF WBC (0.708). The heightened diagnostic efficacy of CI in this context was attributed to its internal instinct, as CI was specifically designed to minimize the confounding effects of blood contamination in CSF analysis, which was crucial in cases where CSF RBC counts were elevated due to surgical trauma or hemorrhage^18^. This feature enhanced its specificity in distinguishing between actual infection and other causes of CSF abnormalities. In the subgroup analysis of Gram-negative bacterial intracranial infection, CSF PCT emerged as a standout biomarker with an impressive AUC of 0.950, which indicated that CSF PCT possessed superior sensitivity and specificity in identifying Gram-negative bacterial infections within the intracranial compartment. Furthermore, the combination of CSF PCT with other biomarkers significantly enhanced diagnostic accuracy. Indeed, PCT had a better predictive value for infections caused by Gram-negative bacteria which produce large lipopolysaccharides (LPS) to stimulate PCT production^28^. Therefore, CI, CSF PCT, and CSF IL-6 alone and their combination demonstrates certain advantages in the diagnosis of intracranial infections, especially in the condition of CSF RBC counts exceeding 3,000 × 10^6^/L or Gram-negative bacterial infections.

Despite no significant difference in 28-day mortality between the two groups in this current study, significant worse outcomes were also observed after intracranial infection evidenced by a longer length of stay in the ICU and higher 6-month GOS scores in comparison with patients without intracranial infection. The lack of significant difference in 28-day mortality between the two groups may be due to our prompt use of mNGS for diagnostic assistance and active intervention immediately after the onset of suspected intracranial infection, which potentially reduced the mortality rate in the infected group, differing from previous research^4;7;8^. Further prospective multi-centre larger sample size studies are needed to further investigate the effect of intracranial infection on the outcomes of patients after neurosurgery.

Several limitations also exist in the current study. First, although improving the diagnosis efficacy of intracranial infections, these indicators like CI, CSF PCT, or CSF IL-6 and their combinations cannot directly indicate exact etiological infection as metagenomic sequencing does. However, integrating these indicators such as CI, CSF PCT, CSF IL-6 or their combinations into routine cerebrospinal fluid tests could better improve the diagnostic efficacy of intracranial infections, which would provide a promising strategy especially in the condition of metagenomic sequencing unavailable in some resource-limited settings. Second, the withdrawal of some centers from this study due to the COVID-19 pandemic may have introduced selection bias, as the sample was restricted to two ICU centers, which limits the generalizability of our findings. Although a substantial sample was included, potential undetected confounding factors and the necessity for statistical adjustments for multiple comparisons were not adequately addressed. A larger cohort study would be required to confirm these results and bolster the conclusions. Meanwhile, whether the patients had intracranial infections other than bacteria could not be completely ruled out. Although some samples had conducted the DNA and RNA gene sequencing, most of the CSF samples had only been performed. Whether these indicators like cell index, CSF PCT, CSF IL-6 or their combinations have diagnostic value for intracranial virus infection after neurosurgery is unknown, which merits further investigation.

In conclusion, CI, CSF PCT, and CSF IL-6 have certain diagnostic value in the early diagnosis of bacterial intracranial infection after neurosurgery, and their combination has more powerful diagnosis efficacy. Of note, the diagnostic value of CI alone or combination is higher when CSF RBC > 3,000 × 10^6^/L, so does the CSF PCT alone or combination when intracranial infection by Gram-negative bacteria. Therefore, our study provides an effective, sensitive and specific diagnostic method for bacterial intracranial infection after neurosurgery, which is worthy of further application.

## Data Availability

The data that support the findings of this study are available upon request due to privacy restrictions. Data are available in a de-identified and aggregated form and can be accessed by qualified researchers who provide a methodologically sound proposal. Requests to access the data should be directed to the corresponding author, Dr. GS Zhang, or the alternative contact person, Dr. QJ Chen.
